# Supplementary material for: Short-Term Adverse Effects Immediately after the Start of COVID-19 Booster Vaccination in Vietnam
Source: Vaccines (Basel). 2022 Aug 16;10(8):1325. doi: 10.3390/vaccines10081325 (PMC9414515; doi:10.3390/vaccines10081325)
Supplement: Supplementary file 1 [file vaccines-10-01325-s001.zip › vaccines-1831417-supplementary.pdf]

Supplementary table S1: Factor associated with the presence of side effects occurring after first dose of COVID-19 vaccine

	Bivariate analysis			Multivariate analysis*		
	OR	95%CI	P-value	Adjusted OR	95%CI	P-value
Age (year)	0.96	0.95 – 0.97	<0.0001	0.97	0.96 – 0.98	<0.0001
Gender						
Female	reference			reference		
Male	0.42	0.31 – 0.58	<0.0001	0.46	0.33 – 0.65	<0.0001
Chronic disease						
No	reference			reference		
Yes	0.46	0.28 – 0.77	0.003	1.11	0.57 – 2.14	0.77
COVID-19 vaccine						
AstraZeneca	reference			reference		
Sinopharm	0.08	0.04 – 0.14	<0.0001	0.09	0.05 – 0.17	<0.0001
Pfizer	0.57	0.31 – 1.05	0.07	0.57	0.31 – 1.05	0.07
Moderna	1.25	0.79 – 1.98	0.33	1.25	0.79 – 1.98	0.33
Sputnik V	NA	NA	NA	NA	NA	NA

*: logistic regression, only variables with p-value <0.2 were introduced in the model; NA: not applicable because only one person was vaccinated with Sputnik V vaccine for the first dose

Supplementary table S2: Factor associated with the presence of side effects occurring after second dose of COVID-19 vaccine

	Bivariate analysis			Multivariate analysis*		
	OR	95%CI	P-value	Adjusted OR	95%CI	P-value
Age (year)	0.99	0.98 – 1.01	0.06	0.99	0.98 – 1.00	0.11
Gender						
Female	reference			reference		
Male	0.48	0.37 – 0.61	<0.0001	0.47	0.36 – 0.61	<0.0001
Chronic disease						
No	reference					
Yes	0.82	0.52 – 1.30	0.40			
COVID-19 vaccine						
AstraZeneca	reference			reference		
Sinopharm	0.37	0.22 – 0.62	<0.0001	0.42	0.24 – 0.72	0.002
Pfizer	3.23	1.77 – 5.89	<0.0001	3.54	1.92 – 6.53	<0.0001
Moderna	8.56	5.00 – 14.68	<0.0001	9.29	5.38 – 16.04	<0.0001
Sputnik V	NA	NA	NA	NA	NA	NA

*: logistic regression, only variables with p-value <0.2 were introduced in the model; NA: not applicable because only one person was vaccinated with Sputnik V vaccine for the second dose

Supplementary table S3: Factor associated with the presence of side effects occurring after booster dose of COVID-19 vaccine

	Bivariate analysis			Multivariate analysis*		
	OR	95%CI	P-value	Adjusted OR	95%CI	P-value
Age (year)	1.01	0.99 – 1.01	0.51			
Gender						
Female	reference			reference		

Male	0.57	0.43 – 0.75	<0.0001	0.57	0.43 – 0.76	<0.0001
Chronic disease						
No	reference			reference		
Yes	1.49	0.83 – 2.68	0.18	1.40	0.76 – 2.56	0.28
COVID-19 vaccine						
AstraZeneca	reference			reference		
Sinopharm	NA	NA	NA	NA	NA	NA
Pfizer	3.25	2.48 – 4.28	<0.0001	3.21	2.43 – 4.22	<0.0001
Moderna	ND	ND	ND	ND	ND	ND

*: logistic regression, only variables with p-value <0.2 were introduced in the model; NA: not applicable because only one person was vaccinated with Sinopharm vaccine for the booster dose; ND: not determined because all persons vaccinated with Moderna vaccine have had a side effect after their booster dose.
